# Allelic Variants for Candidate Nitrogen Fixation Genes Revealed by Sequencing in Red Clover (*Trifolium pratense* L.)

**DOI:** 10.3390/ijms20215470

**Published:** 2019-11-02

**Authors:** Oldřich Trněný, David Vlk, Eliška Macková, Michaela Matoušková, Jana Řepková, Jan Nedělník, Jan Hofbauer, Karel Vejražka, Hana Jakešová, Jan Jansa, Lubomír Piálek, Daniela Knotová

**Affiliations:** 1 Agricultural Research, Ltd., Zahradní 1, 664 41 Troubsko, Czech Republic; matouskova@vupt.cz (M.M.); nedelnik@vupt.cz (J.N.); hofbauer@vupt.cz (J.H.); vejrazka@vupt.cz (K.V.); 2 Department of Experimental Biology, Masaryk University, 625 00 Brno, Czech Republic; Vlk.DavidR@email.cz (D.V.); mackova.e.94@gmail.com (E.M.); repkova@sci.muni.cz (J.Ř.); 3 Red Clover and Grass Breeding, 724 47 Hladké Životice, Czech Republic; hana.jakesova@tiscali.cz; 4 Institute of Microbiology of the Academy of Sciences of the Czech Republic, 142 20 Prague, Czech Republic; jansa@biomed.cas.cz; 5 Department of Zoology, Faculty of Science, University of South Bohemia, 370 05 České Budějovice, Czech Republic; lpialek@yahoo.com; 6 Research Institute for Fodder Crops, Ltd., 664 41 Troubsko, Czech Republic; knotova@vupt.cz

**Keywords:** associated genes, associated polymorphisms, genome-wide association, biological nitrogen fixation, red clover

## Abstract

Plant–rhizobia symbiosis can activate key genes involved in regulating nodulation associated with biological nitrogen fixation (BNF). Although the general molecular basis of the BNF process is frequently studied, little is known about its intraspecific variability and the characteristics of its allelic variants. This study’s main goals were to describe phenotypic and genotypic variation in the context of nitrogen fixation in red clover (*Trifolium pretense* L.) and identify variants in BNF candidate genes associated with BNF efficiency. Acetylene reduction assay validation was the criterion for selecting individual plants with particular BNF rates. Sequences in 86 key candidate genes were obtained by hybridization-based sequence capture target enrichment of plants with alternative phenotypes for nitrogen fixation. Two genes associated with BNF were identified: ethylene response factor required for nodule differentiation (*EFD*) and molybdate transporter 1 (*MOT1*). In addition, whole-genome population genotyping by double-digest restriction-site-associated sequencing (ddRADseq) was performed, and BNF was evaluated by the natural ^15^N abundance method. Polymorphisms associated with BNF and reflecting phenotype variability were identified. The genetic structure of plant accessions was not linked to BNF rate of measured plants. Knowledge of the genetic variation within BNF candidate genes and the characteristics of genetic variants will be beneficial in molecular diagnostics and breeding of red clover.

## 1. Introduction

The family Fabaceae, consisting of more than 750 genera and 19,000 species, is the third largest family of flowering plants and, in terms of agricultural importance, the second most important family, after Poaceae. Several species from this family serve as genetic model organisms (e.g., *Medicago truncatula* Gaertn., *Pisum sativum* L., and *Lotus corniculatus* L.). One of the largest genera of the Fabaceae family is the clover genus, *Trifolium* L., with more than 250 species [1,2]. This herbaceous genus, which acquired its name as a reference to the characteristic form of the leaf usually consisting of three leaflets (trifoliolate), includes both annual and perennial species and occurs natively in temperate and subtropical regions of the northern and southern hemispheres [3]. The importance of the genus *Trifolium* lies in its agricultural utilization. In addition to several species being cultivated extensively as fodder plants (such as *T. pratense* L., *T. repens* L., *T. hybridum* L., and *T. resupinatum* L.), fast-growing clovers are sown as green manure crops or mixed intercrops to enhance soil fertility and sustainability [4]. As typical for the majority of leguminous plants, *Trifolium* species can establish a mutualistic relationship with the root-nodulating bacteria *Rhizobium leguminosarum* bv. *trifolii*. This initiates a complex process of biological (atmospheric) nitrogen fixation (BNF). In this relationship, the plant provides the bacteria a source of carbon and energy, in addition to phosphorus and other mineral nutrients and also anoxic shelter, and the bacteria supply the plant with nitrogen acquired from the atmosphere, converted into organic compounds utilizable in plant metabolism [5]. BNF in legumes constitutes an irreplaceable nitrogen source for both ecosystems and circulation in nature. Soil N enrichment due to effective BNF is environmentally more sustainable than application of synthetic N fertilizers depending on utilization of nonrenewable sources of energy.

Interactions between nodulating bacteria and root plant systems are highly specific. In many cases, some variant of bacterial strain, or biovar, is able to create functional nodules with only one or several plant species [6] and, to a considerable extent, this determines the efficiency of nodulation and nitrogen fixation [7]. In addition to complexities due to this high specificity, plant breeding directed to the enhancement of nitrogen fixing ability is further complicated by the complexity of the phenotypic trait, as it involves an estimated several hundred genes in nodulation and nitrogen fixation [8]. Red clover (*T. pratense*), with a reported BNF level in aboveground plant tissues as great as 373 kg N∙ha^−1^∙year^−1^ [9], and other *Trifolium* species having high rates of BNF heritability [10] are promising for purposes of plant breeding directed to enhancing nitrogen fixing rates.

Cloned and characterized genes responsible for symbioses are involved in recognition of rhizobial nodulation signals, early symbiotic signaling cascades, infection and nodulation processes, and regulation of nitrogen fixation [8]. Plant–bacteria interaction is initiated by phenolic compounds exuded by the plant rhizosphere and which attract rhizobacteria present in soil. Moreover, these phenolic compounds bind to bacterial transcriptional regulator nodD and induce activation of *nod* genes [11]. Products of *nod* genes, termed nodulation (Nod) factors, are lipochitooligosaccharide signaling molecules. Nod factors are specifically bound to receptors on the root surface inducing morphological alteration and activation of root-specific cascades that enable initiation of nodulation, whereby specific kinases and transcription factors participate [12,13,14]. The systemic signals enable plants to control the number of nodules they form depending upon the number of existing nodules and availability of soil nitrogen [15,16].

Morphological alteration of the root surfaces includes both induction of cell division and curling of root hair to enable bacterial infection of the plant. Infection continues with the creation of an infection thread, which enables bacterial invasion into the cells of the inner cortex. The invasion is followed by activation of gene expression in inner cortex cells, promoting nodule formation and development [17]. The process of nitrogen fixation is performed by a nitrogenase enzyme complex encoded by bacterial *nif* genes [5]. Because reduction of atmospheric nitrogen to ammonia is associated with high energy consumption, it is limited by the availability in soil of phosphorus, a critical component of adenosine triphosphate (ATP). Nitrogenase is extremely sensitive to oxygen exposure, with even low concentrations resulting in irreversible denaturation. To supply bacteria with oxygen for the respiration process while at the same time keeping nitrogenase protected from denaturation, a hemoprotein called leghemoglobin carries oxygen to the peribacteroid membrane while allowing a nearly anaerobic environment to be maintained inside bacteroids [18,19].

Using both forward (mostly chemical mutagenesis) and reverse genetics approaches (such as insertional mutagenesis or gene silencing), some of these genes were already identified. Mostly, this was in model organisms *M. truncatula* and *L. japonicus* [8,20,21,22,23,24,25], thus enabling scientists to search for orthologous genes in other legumes, including *T. pratense*. Using DNA markers and genetic mapping, candidate legume genes likely participating in different signaling pathways were gradually identified by Cregan et al. [26], Santos et al. [27,28], and Nicolás et al. [29] in the model crop soybean. Recently, key regulating genes conferring nodulation and nitrogen fixation were revealed using comparative genomic and transcriptomic analyses, mainly in *Pisum sativum* L., *Glycine max* (L.) Merr., and *Phaseolus vulgaris* L. [30,31,32].

Searching for genes involved in symbiotic nitrogen fixation in red clover is facilitated by its small genome size (estimated 418 Mb [33]). Genomic data are available for two varieties, tetraploid Tatra (∼314.6 Mbp [34]) and diploid Milvus (∼309 Mbp [35]). Both were recently de novo sequenced using next-generation sequencing (NGS). The acquired genome sequences were subsequently annotated, resulting in annotation of 47,398 protein-coding genes from 64,761 predicted genes in variety Tatra [34]. Moreover, several gene families characteristic for red clover were revealed, including 11 leghemoglobin genes and 542 nodule-specific cysteine-rich peptides [34]. For the variety Milvus, 22,042 of a total 40,868 annotated genes were located on seven pseudomolecules (chromosomes) and, using *M. truncatula* as a reference sequence [36], a physical map was constructed.

NGS methods enable genome-wide mining of DNA polymorphisms associated with the traits analyzed. Genome-wide association studies with high-throughput genotyping by sequencing to identify loci associated with nitrogen fixation efficiency were applied in legumes such as *M. truncatula* [37,38] and soybean [39].

Reduced-representation NGS-based genotyping methods, such as double-digest restriction-site-associated sequencing (ddRADseq) [40], were also proven to be beneficial for detecting genome-wide allele frequency fingerprints [41] of populations. Allelic variants such as single-nucleotide polymorphisms (SNPs) and insertion/deletion variations (InDels) make it possible to reveal genetic structure, identify population-specific variants, and find genotype–phenotype associations. Not only do NGS methods allow for genome-wide study, they also look into sequences within every particular candidate gene using bulk target sequencing approaches, such as hybridization-based sequence capture (SeqCap) target enrichment [42].

Here, we describe within- and between-population variability in nitrogen fixation capacity in red clover and demonstrate the utility of several NGS methods for the purpose of key genes and population genotyping. Our present study was based on two sequencing methods, SeqCap using a hybridization-based strategy and ddRADseq. Our goals were to (i) characterize variability that appears in nitrogen fixation candidate genes in red clover populations, (ii) assess this variability in the context of nitrogen fixation efficiency in various red clover accessions, (iii) analyze how level of variance in host candidate genes explains efficiency of biological nitrogen fixation, and (iv) identify allelic variants present in red clover populations and associated with nitrogen fixation level.

## 2. Results

### 2.1. Nitrogen Fixation Assays

**Figure 1:**
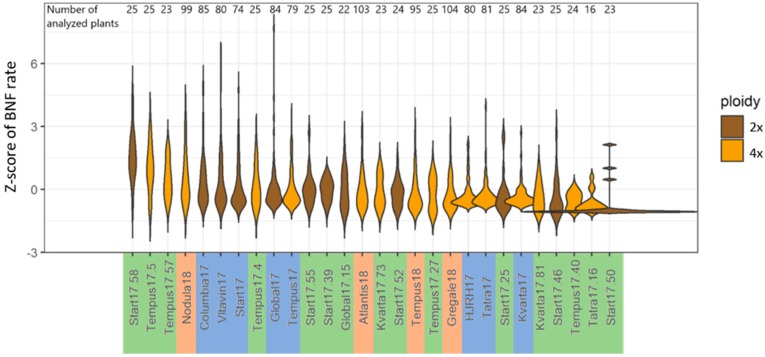
Distribution of *Z*-score for nitrogen fixation rate evaluated in red clover plants using acetylene reduction assay. On the *x*-axis, genotypes are ordered by mean values of nitrogen fixation. Diploid (brown) and tetraploid (yellow) red clover plants were measured in three sets: Set 1 (blue labels with suffix 17), Set 2 (orange labels, suffix 18), and Set 3 (green labels, suffix 17.xx—progeny of selected contrasting plants from Set 1).

In total, 1426 individual plants of 12 diploid and 16 tetraploid accessions were measured in three sets using an acetylene reduction assay (ARA). The characteristics of the intrapopulation distribution of nitrogen fixation level depended on the genotypes of the population (Figure 1).

There were significant differences in BNF rates among accessions within all three plant sets (Table 1). In Set 1, approximately 80 plants per accession were without extreme values of fixation. The Columbia17 accession with the highest nitrogen activity differed significantly (*p* < 0.01) from accessions HJRH17 and Kvarta17 (Table 1). In Set 2, approximately 100 plants per accession were evaluated. From Sets 1 and 2 together, accession Nodula18 was the best fixator according to the mean value of BNF rate, which was among the four highest mean values across all accessions (Figure 1). Remaining accessions only showed nitrogen fixation values near the mean. Progeny of eight high- and eight low-BNF plants from Set 1 were retested in Set 3 (Figure 1; Suffix 17.xx). There were significant differences in BNF level among the offspring both of high and low fixators. Examining more closely the progeny of high fixators, multiple comparison revealed significant differences (*p* < 0.01) between Start17.58 and nine accessions and between Tempus17.5 and four accessions. Among progeny of low fixators, significant differences were confirmed between Start17.50 and six accessions (Table 1). As visible in the Figure 1 violin plot, there exist individual plants in most populations that are highly effective BNF rate outliers with several times greater fixation efficiency relative to others.

For ARA validation, we included measuring of an ethylene standard and measuring the same accession in two consecutive years. The regularly measured ethylene control varied little. Coefficients of variation of standardized ethylene control (97.5 ppm) measurement were 12.5%, 5.6%, and 5.2% in sets 1, 2, and 3, respectively. Tempus plants were planted in both Set 1 (79 plants) and Set 2 (95 plants) as a control variety for nitrogen fixation measurement. In both years of analysis, the results for Tempus accession were similar; comparison of the two plant collections showed no statistically significant differences (Table 1), and both mean values of nitrogen fixation (Tempus17 and Tempus18) were in the middle part of the distribution plot (Figure 1).

### 2.2. Candidate Gene Target Sequencing

Two panels of selected BNF candidate genes were compiled, and DNAs from plants with contrasting BNF level were sequenced. Panel 1 contained 17 genes with key roles in BNF studied on a model organism (Supplementary Table S1). In this panel, 24 high-BNF and 24 low-BNF plants (Supplementary Table S2) were selected according to ARA and then sequenced. The number of polymorphisms per candidate gene varied between 220 and 887. Polymorphisms were associated with BNF phenotypes while correcting for genetic structure and plant kinship.

**Figure 2:**
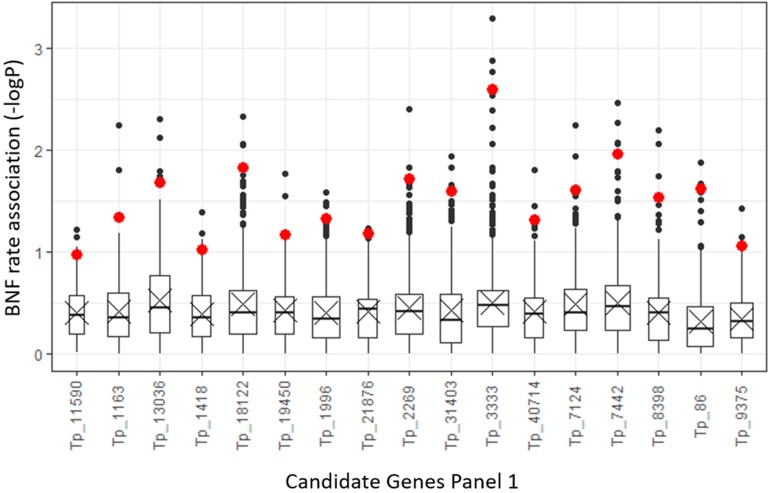
Boxplot of −logP association values for Panel 1 of nitrogen fixation candidate gene polymorphisms based on mixed linear model incorporating both population structure and relationships among accessions. Red dots show mean values for the 10 highest −logP values of each associated polymorphism. The highest mean value was that of Tp_3333, which is the sequence with the ethylene response factor required for nodule differentiation (*EFD*) gene [35].

The gene ethylene response factor required for nodule differentiation (*EFD*) from the ethylene response factor (ERF) family that was found in targeted sequence Tp_3333 had the most closely associated polymorphisms (Supplementary Table S3) with BNF phenotypes in Panel 1 (Figure 2).

Panel 2 consisted of 69 candidate genes, which were predominantly selected according to literature specifications with prevalent expression in *M. truncatula* nodules [43]. DNA samples from 25 high-BNF and 25 low-BNF tetraploid plants were sequenced (Supplementary Table S2). Coverage along capture sequences varied among samples (Supplementary Figure S1). Gene polymorphisms were called with high quality and homogenously along the sequences due to the sufficient coverage.

**Figure 3:**
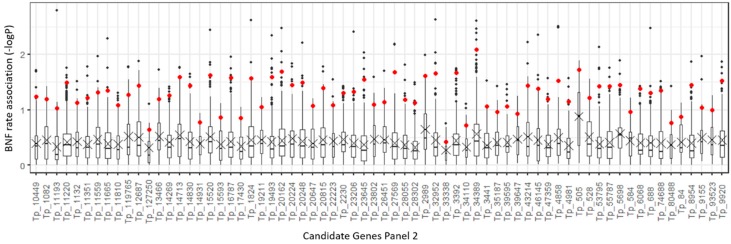
Boxplot of −logP association values for Panel 2 of nitrogen fixation candidate gene polymorphisms based upon a mixed linear model incorporating both population structure and relationship between accessions. Red dots show mean values for the 10 highest −logP values of each associated polymorphism. The highest mean value was for Tp_34389, which is the sequence carrying the molybdate transporter type 1 (*MOT1*) gene [35].

The number of polymorphisms ranged from 18 to 696 per candidate gene sequence. The gene coding molybdate transporter type 1 (*MOT1*) on targeted sequence Tp_34389 was evaluated as having strong effect on the BNF phenotype. This was proven by the highest mean *p*-value among 10 polymorphisms (Supplementary Table S3) with the highest association levels (Figure 3). *MOT1* [35] plays a key role in the BNF process, and its main function is to provide molybdenum for synthesis of the iron–molybdenum cofactor of nitrogenase [44].

**Figure 4:**
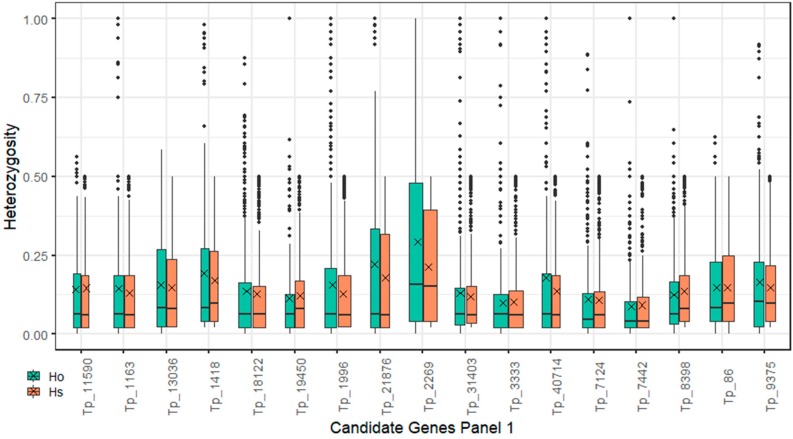
Boxplot of observed (Ho; blue boxes) and expected (Hs; orange boxes) heterozygosity of Panel 1 nitrogen fixation candidate genes. Hs expresses the level of genetic variability. Crosses indicate mean values. Horizontal lines in boxes indicate medians. Bottoms and tops of boxes indicate the first and third quartiles of the dataset. Whiskers indicate range of data but the maximum length of each is 1.5 times greater than the height of its box. Remaining points are outliers. The boxes are drawn with widths proportional to the square roots of the numbers of polymorphisms in targeted sequences.

**Figure 5:**
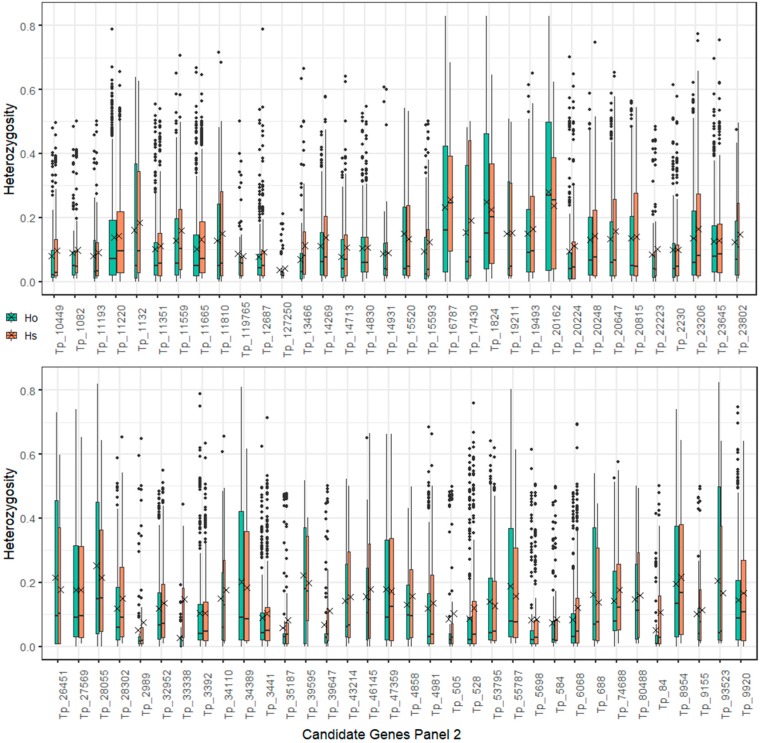
Boxplot of observed (Ho; blue boxes) and expected (Hs; orange boxes) heterozygosity of Panel 2 nitrogen fixation candidate genes. Hs expresses the level of genetic variability. Crosses indicate mean values. Horizontal lines in boxes indicate medians. Bottoms and tops of boxes indicate first and third quartiles of the dataset. Whiskers indicate range of data but the maximum length of each is 1.5 times greater than the height of its box. Remaining points are outliers. The boxes are drawn with widths proportional to the square roots of the numbers of polymorphisms in targeted sequences.

Expected heterozygosity (Hs) was used as a criterion for assessing diversity levels of candidate genes alleles. In the candidate genes of Panel 1, the sequences with the three highest mean Hs values were Tp_2269 with the gene nod factor perception (*NFP*), Tp_21876 with the gene partner of NOB1-like (*PNO1-like)*, and Tp_1418 with the gene cytokinin response 1 (*CRE*) cytokinin receptor kinase/nodule organogenesis (Figure 4). In any of the candidate genes of Panel 1, there was no obvious difference between the expected and observed heterozygosity found. In the candidate genes of Panel 2, the two targeted sequences with the highest level of diversity (Hs = 0.23) were Tp_16787, which encodes the gene for nuclear transcription factor Y subunit C2 (NF-YC2), and Tp_20162, encoding flotillin (FLOT) protein. The means of both genes were shown to be close to their medians, indicating symmetrical distribution of their Hs values (Figure 5). In comparison with other genes of Panel 2, there was an obvious difference found between the expected and observed heterozygosity in two of the sequences with candidate genes (Tp_33338 and Tp_84). We found significantly higher values of the expected heterozygosity than values of the observed heterozygosity (*p* < 0.01) in both of the genes using a Mann–Whitney U test. The gene coding *MOT1* on targeted sequence Tp_34389, which manifested the strongest association with the BNF rate phenotype, had a modest diversity level (Hs = 0.164). Among targeted sequences with small diversity were Tp_127250 with the gene non-symbiotic hemoglobin 2 and Tp_2989 with the gene rac-like GTP-binding protein (*ARAC10*). These targeted sequences had low numbers of polymorphisms with low mean Hs, thus implying conserved region and importance of the genes (Figure 5). From seven targeted sequences for leghemoglobins, we could distinguish three groups. Sequences Tp_119765 and Tp_127250, with leghemoglobins genes, were in the first group having low polymorphism counts with low diversity. Sequence Tp_93523, with a leghemoglobin gene, had a low polymorphism count but the highest diversity level among leghemoglobin genes. Leghemoglobin sequences from the third group (Tp_1132, Tp_13466, Tp_14713, Tp_3441) had high polymorphism counts per sequence with medium genetic diversity levels.

### 2.3. ddRADseq and N Isotopic Composition

**Figure 6:**
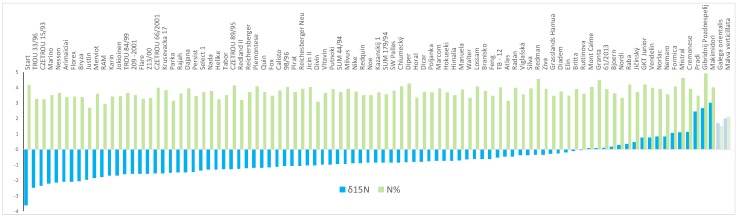
Interpopulation diversity of biological nitrogen fixation as revealed by natural ^15^N abundance measurement of red clover leaves (δ^15^N values are shown in blue). Alongside, N concentrations in the leaves are displayed in green (weight %). The control non-nitrogen symbiotic plant (*Malva verticillata*) and leguminous plant *Galega orientalis* uninoculated by symbiotic partner are located on the right side.

In addition to the targeted sequencing approach that assesses variability of BNF key genes, we harnessed the power of high-throughput sequencing to assess complex whole-genome genotype. Ninety-one *T. pratense* diploid accessions were genotyped at population level using the ddRADseq approach and were phenotypically analyzed for N isotopic composition (indicative of BNF) using the natural ^15^N abundance method, using isotope ratio mass spectrometry (Figure 6). The first three accessions with the highest BNF level were the variety Start and two wild accessions, TROU 33/96 and CZETROU 15/93. N concentration was measured together with isotope composition. No obvious correlation between isotope composition and N concentration in the leaves was found.

Altogether, 91,589 polymorphisms (Supplementary Table S4 were identified with a maximum of 50% missing information, and the minor allele occurred for more than 5% of samples. Sixty-one percent of polymorphisms were mapped to seven linkage groups on the red clover reference genome and 39% of them were mapped to the remaining contigs. The mean coverage of polymorphism was 39.7 × per accession. The mean Hs of polymorphisms was 0.23, which points to a high level of diversity in *T. pratense* populations and corresponds to red clover’s cross-pollination system.

**Figure 7:**
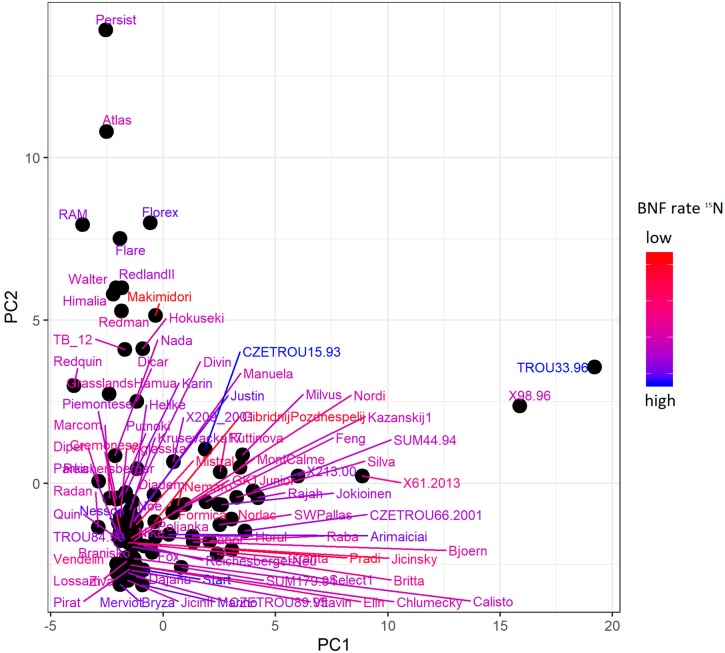
Principal component analysis (PCA) plot of genetic structure of genotype data using 91 samples from red clover populations. PC1 and PC2 indicate principal components. Color scale shows delta ^15^N value that corresponds to biological nitrogen fixation (BNF) level (red color indicates low BNF level, blue indicates high BNF level).

In order to assess genetic diversity and its comparison to BNF level, principal component analysis (PCA) was performed. The first two principal components (PCs) of the PCA (Figure 7) explained just 5.6% (3.0% and 2.6% for PC1 and PC2, respectively) of genotypic variance. Despite the weak determination of variance by the first and second PCs, they did distinguish a basic pattern of genetic diversity among the accessions. While the first PC separated in particular wild-type accessions, the second separated varieties. The rest of the accessions formed the main group. Evidently, BNF level did not correspond with this main diversity pattern in the first two PCs, although accession TROU 33/96, which had the second highest BNF rate, was genetically the most different from the others according to the first PC. Moreover, correlation analysis of other PCs up to PC30 revealed no strong correlation level between any genetic structure pattern and phenotype (Supplementary Figure S3), although some PC correlations did show closer relationships with phenotype in comparison with those of other PCs.

**Figure 8:**
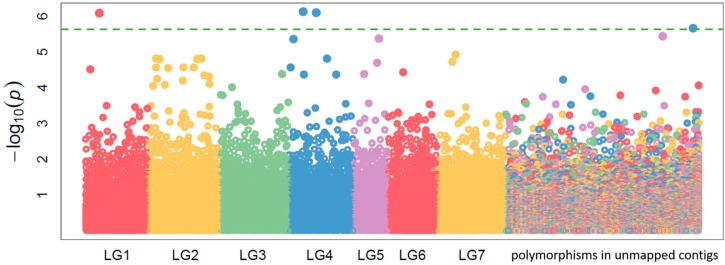
Manhattan plot of genome-wide association of allele frequency and ^15^N nitrogen fixation phenotypes using the FarmCPU algorithm. The different colors (LG1–LG7) indicate different linkage groups [35]. The segment to the far right shows the polymorphisms unmapped to the linkage groups. The green line indicates the false discovery rate-adjusted *p*-value of 0.05 using the Benjamini–Hochberg correction [46].

In order to find associations between genotype and phenotypes, an association study was conducted using the FarmCPU algorithm [45]. We identified three SNPs and one InDel variant that were significantly associated with BNF phenotype (Figure 8) (false discovery rate-adjusted *p*-value < 0.05). Two SNPs lay on linkage group 4 (LG4), one InDel lay on LG1, and one SNP lay on an unmapped contig. Some of the variants were located near genes with functions in the BNF process (Supplementary Table S3). The first mapped significant associated SNP was identified in LG4 position 6,307,333 bp within an intergenic region between genes annotated as mitochondrial rho GTPase 1-like protein and auxin response factor and near the gene for sulfotransferase. The second associated mapped SNP, in LG4 position 12,136,158 bp, lay in an exon of an uncharacterized protein in the neighborhood of two ethylene-responsive transcription factor 3-like genes. The InDel positioned on LG1 at 6,268,253 bp was located in an intron of the gene for lipid phosphate phosphatase 2-like protein and near to several genes for amino-acid permease BAT1-like protein. The third associated SNP had an association level very close to the threshold of association and lay on unmapped contig FKJA01001578.1 at 124 bp, near to the gene for transcription factor DIVARICATA-like protein.

In order to assess the proportion of the total variance explained by the genetic variance, we estimated marker-based narrow-sense heritability from genotype polymorphisms and ^15^N BNF rate phenotype data. The ^15^N BNF rate-estimated heritability was 84.7%.

### 2.4. Polymorphism Annotations

**Figure 9:**
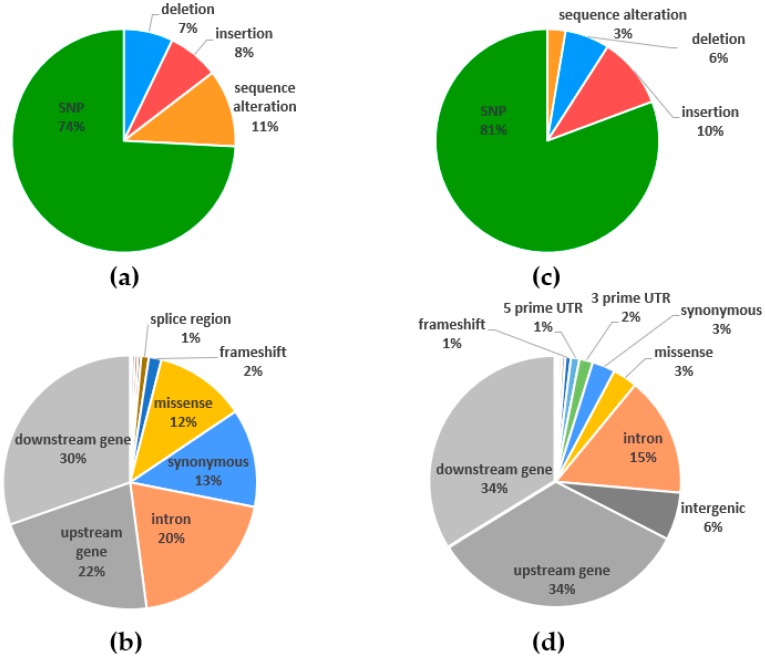
Polymorphism annotation of candidate genes for nitrogen fixation in red clover evaluated by hybridization-based sequence capture (SeqCap) (**a**,**b**) and whole-genome population genotyping double-digest restriction-site-associated sequencing (ddRADseq) (**c**,**d**). Distribution of polymorphism classes (**a**,**c**) and polymorphism consequences (**b**,**d**).

Annotation of variants in candidate genes for BNF (Figure 9a,b) and whole-genome population genotyping (Figure 9c,d) were obtained. SNPs were revealed as the most frequent variants. Other variants resulted from length differences (deletion and insertion), and the rest of the variants were based on sequence alterations (Figure 9a,c). In target sequencing of Panels 1 and 2, we found a greater part of sequence alterations than in ddRADseq population genotyping. From the perspective of consequences, half of the variants from targeted sequencing belonged to genic regions (Figure 9b), while ddRADseq population genic variants (Figure 9d) formed only one-quarter of the total variants. Variants of Panel 1 were 29% from genic regions in comparison with variants from Panel 2 that constituted 60% of genic variants. Consequently, Panel 1 was focused on the sequencing of 17 candidate genes and their broad surroundings, but Panel 2 was focused on a higher number of genes and their near-adjacent sequences. Missense variants formed a similar part of variants, as did synonymous variants under both genotyping approaches. For targeted sequencing and ddRADseq population genotyping, we identified a minority of genic variants, such as frameshifts (2% and 1%, i.e., 491 and 431, respectively; Figure 9b,d), stop gained (122 and 160), stop lost (27 and 17), and start lost variants (13 and 16), with severe impact on gene expression.

### 2.5. Validation of Selected InDel Polymorphisms

Length of validated InDels obtained by targeted sequencing ranged from 9 to 289 bp. From 10 designed primer pairs (Supplementary Table S5), nine gave specific products. The primer for InDel in position 5325 within the *NSP2* gene (targeted sequence Tp_7442) generated no product, but its existence was demonstrated by another primer pair. Analysis of the targeted sequence Tp_19450 with the defective in nitrogen fixation (*DNF2)* gene confirmed the existence of an InDel, but its length was about 200 bp longer than the expected length. Analysis of the remaining InDels confirmed their existence and validated the sequencing data. The lengths of the amplified products were in line with those of the expected products (Supplementary Figure S2).

## 3. Discussion

BNF is a complex process wherein many genes participate along with the context of environmental conditions [47]. The potential amounts of nitrogen that can be fixed are several times greater than the amounts of nitrogen usually fixed in the fields. The amount of nitrogen fixed by legume–rhizobia symbioses may be increased by as much as 300% through plant breeding and crop management [48]. The potential that plant selection for symbiotic activity may be highly effective is also supported by the data on high heritability. In a relatively stable field environment, the broad-sense heritability of nodulation traits in soybean may exceed 0.8 [47,49], suggesting that nodulation traits are mainly controlled by genetic loci and are useful for breeding varieties with high BNF capacity. In *Trifolium incarnatum* inbred lines, broad-sense heritability was estimated to be similarly high (up to 0.91) [50].

Although the genic nature of BNF efficiency is undeniable, it is the complexity and difficulty of phenotyping that prevented the breeding of red clover for BNF efficiency from being accomplished successfully [51]. With the availability of high-throughput target and genome-wide genotyping approaches, however, new ways were opened for dealing with complex polygenic traits. Recent omics studies revealed deep complexity of the nitrogen fixation process.

Various legume species perform differently in fixing nitrogen, and interspecies variability is well known [10,52]. A study comparing fixation efficiency between model plant *M. truncatula* and fodder crop *M. sativa* showed several-fold lower efficiency in *M. truncatula* than in *M. sativa* [53]. Significant intraspecific variability in BNF efficiency in red clover was frequently observed and evaluated, and phenotypic variability does not appear to be related to ploidy level [54,55]. Here, we evaluated intraspecific variability in symbiotic activity and BNF capacity in red clover, and two methods were applied, indirect (acetylene reduction; ARA) and (isotopic; ^15^N) estimation of nitrogenase activity [56,57]. ARA was an effective criterion for red clover populations and selection of individual plants with high rates of fixation. Based on ARA of nearly 1500 red clover plants, we observed differences among varieties and among individuals within a variety. The distribution of actual fixation level had a specific characteristic. The largest proportion of plants had low fixation efficiency up to the mean level, while a smaller proportion of plants had higher efficiency, but nearly all of the plants were outperformed by a couple of plants having fixation efficiency several times greater than the mean value of the measured population. This was seen mainly in default populations from Sets 1 and 2. Populations from Set 3 were influenced by selection and, therefore, interpopulation variability in Set 3 was also the highest.

Our research highlights that the breeding value of a plant should be based on progeny performance, and especially so in self-sterile species such as red clover when breeding for a trait as complex as BNF efficiency. Three populations—Start17.58, Tempus17.5, and Tempus17.57 (Figure 1)—were evaluated as being the best fixators among progeny of the selected best BNF plants from Set 1. Even though Start17.58 and Tempus17.5 were the offspring from high-BNF rate plants from Set 1, population Tempus 17.57 was the offspring from low-BNF rate selected plants from Set 1. This confirms the need to select plants based on progeny performance, which is feasible due to the perennial character of red clover. All other red clover population studies showed mostly plants with low fixation rate and rarely plants with high fixation rate. Outlier plants that outperformed the others contributed greatly to the population mean BNF level, but it is probably not achievable to select a population consisting solely of superior plants on the highest performance level. Superior plants occurred in most populations, and, in addition to the additive effect of many genes, their superiority can be derived also from non-additive effects such as a heterotic effect [51]. A high nitrogen fixation rate was confirmed for the Nodula accession, a variety bred for high BNF efficiency. In accord with our previous experience with the Columbia accession, it stood among the most BNF-efficient genotypes. This study’s results in Columbia17 confirmed this disposition. The idea of breeding for nitrogen fixation efficiency is limited by the cost of BNF for plants. Leguminous plants have an effective mechanism for holding BNF at the right level [58].

Different types of BNF evaluation methods with many variations were designed [52], but the ARA and natural ^15^N-abundance methods are commonly used. Each method has its own advantages and difficulties that must be considered. ARA was the subject of much discussion because many factors influence BNF rate, such as temperature [59] and light [60], but uniform measurement conditions allow the relative assessment of BNF rate [61]. ARA is focused on instantaneous measurement, and it is suitable for the comparison of actual BNF levels in specific time. The natural ^15^N-abundance method is time-integrated, and it inherently assesses the total amount of N fixed for the sample growth period. The natural ^15^N-abundance method is, therefore, appropriate when we assess interpopulation BNF rate, because we can filter out the influence of actual environment–genotype interaction (such as phenological stages). It assesses a total growth period, whereas ARA is suitable for high-throughput selection in populations where genetic differences among plants are smaller and we can perform selection based on relative comparison. The difference between these two methods is one of the factors why the results of genome-wide associations do not correspond with the results of association of candidate genes. Another reason for non-corresponding results could be that target sequencing analysis was focused on individual samples within the population, while the genome-wide association sample set consisted of bulked population samples where the genotype was expressed as the allelic frequency in a population.

The natural ^15^N-abundance method was applied for a collection of diverse populations. The populations with the second and third highest fixation rate (Figure 6) were wild accessions. This was in agreement with Provorov and Tikhonovich [10], who concluded that symbiotic potential in the wild-growing (local) varieties is usually greater than that in commercial varieties. Not all of the wild accessions belong to the best nitrogen fixators, however, because there is BNF rate variation within wild accessions, as well as within cultivated accessions. The same conclusion arose from assessment of the ^15^N BNF rate and genetic structure. The ^15^N BNF rates were not clearly distributed according to the first two PCs of the PCA that corresponded to the main genetic pattern of collection (Figure 7); however, the unequal correlation level between other PCs and the BNF rate (Supplementary Figure S3) suggests low but possible influence of genetic structure on BNF rate. The best nitrogen fixator was the variety Start, which was also a progenitor of the highest BNF level populations in Set 3. Both natural ^15^N-abundance and ARA methods confirmed the Start variety to be appropriate default material for BNF rate selection.

The complexity of genetic control over BNF corresponds to the complexity of the symbiotic BNF process. The contact among plants and bacteria precedes the establishment of a successful symbiosis. The host plant must discern the right partner within the soil biome. It must distinguish and select the rhizobia partner from pathogens and also from among distinct rhizobia species and inappropriate strains. Successful infection is followed by nodule organogenesis. Both processes are driven and regulated by orchestration of gene expression. More than 4000 differentially expressed transcripts were identified in nodules and roots, and more than 500 transcripts were exclusively detected in nodules of the model organism *M. truncatula* [43]. Red clover, a non-model organism, is a significant fodder crop whose breeding for high nitrogen fixation capacity would be valuable, without molecular approaches, albeit difficult and slow. BNF seems to be a polygenic trait [51] that is based on a couple of essential genes [58] that are themselves modulated by many genes with a potential effect on BNF rate [43]. We took the first steps to identifying red clover key genes playing central roles in the formation of root nodules and nitrogen fixation variability. We used an association study based on hybridization-based sequence capture target enrichment and a genome-wide approach, focused on finding variants and genome locations where genetic variance meets phenotype variance and they influence one another.

One of the genes having strong polymorphism association with BNF that arose from the analysis of candidate genes Panel 1 was ethylene response factor required for nodule differentiation (*EFD*). This gene belongs to the ethylene response factor (ERF) family that is a part of the AP2/ERF superfamily (containing the APETALA2 DNA binding domain) [62,63]. The ERF gene family includes plant-specific transcription factors that play roles in response to biotic and abiotic stress, control of organ development, and cell division and differentiation [62,64]. *EFD* is located in the nucleus. It is most expressed in nodule primordia and at the border of infection zones I and II. *EFD* activity is probably not induced by ethylene. The *EFD* role in nodule development and differentiation is dual. *EFD* negatively regulates the nodulation process, affecting the number of infections, but *EFD* also positively influences bacterial and plant cell differentiation in the late stages of nodule development. It was detected in mutant *efd-1* plants, for example, where it causes a later onset of nodule senescence. *EFD* also plays a role in regulation of the pathway of cytokines that influence nodule meristem activity [64].

The analysis of candidate genes in Panel 2 revealed another gene strongly associated with BNF, the molybdate transporter 1 [35]. Molybdenum is an essential plant micronutrient involved in nitrogen fixation and in some other plant enzymatic processes like nitrate assimilation, phytohormone biosynthesis, purine metabolism, sulfite detoxification, and amidoxime reduction [65]. Molybdenum is present in soil in the form of oxyanion molybdate, and the intake of this nutrient is managed by molybdate transporters. The molybdate transporter type 1 family is involved in molybdate transport to the cytoplasm of nodule cells. These transporters are located in the plasma membrane of infected and uninfected cells within the interzone and early fixation zone of the nodule. From the cell cytoplasm, molybdate must be transported across the symbiosome membrane. This transport is presumed to be performed by the symbiotic sulfate transporter SST1 [66], after which ATP-binding cassette transporter (ModABC) transfers molybdate into the bacteroid [67,68]. The molybdenum in a plant cell is a component of the iron–molybdenum cofactor (FeMoco) of nitrogenase. In knockout *M. truncatula* line mot1.3-1, lower nitrogenase activity and reduced plant growth as a result of a lack of nitrogen were observed. Under non-symbiotic conditions, *M. truncatula* plants showed no physiological or phenotypical difference from a control group, and this result was consistent with a hypothesis that the MOT1 transporter is evolutionarily specialized to provide molybdenum for symbiotic nitrogen fixation [44].

A part of the analysis of candidate genes in Panel 2 was an analysis of leghemoglobin genes. Leghemoglobin proteins play an important role in the activity of the oxygen labile enzyme nitrogenase [69]. Leghemoglobins maintain the low free oxygen level in the nodule-infected zone [70], and they also transport oxygen to sites of respiration, thus enabling ATP production in a low-oxygen environment [71]. In *M. truncatula*, genes for leghemoglobin are among the most strongly expressed genes in nodule tissue [72]. Ištvánek et al. [34] identified in red clover a similar number of leghemoglobin genes as found in *M. truncatula*. The number of nine leghemoglobin genes in red clover coincides with the number in *M. sativa*. The family of non-symbiotic hemoglobin genes shows only limited amino-acid sequence similarity to the symbiotic hemoglobins. Genes encoding this type of hemoglobin were cloned from the nitrogen-fixing species [73] and from plants that do not fix nitrogen, including monocots [74] and *Arabidopsis thaliana* [75]. These non-symbiotic hemoglobins are typically expressed at low levels in roots and leaves [76,77]. Functions of non-symbiotic hemoglobins are not yet clearly understood [78], although they may play a role in plant survival by increasing the energy status of the cells under hypoxic conditions [79,80]. Seven genes for leghemoglobins were analyzed as a part of candidate gene Panel 2. They can be distinguished into three groups according to the levels of their genetic diversity.

Target sequencing of BNF candidate genes of plants with alternative phenotypes for nitrogen fixation and whole-genome population genotyping using ddRADseq demonstrated two complementary methods for using knowledge about known key genes from related model organisms and simultaneously assessing whole-genome genotype information to exploit complex genetic information from species of interest. Polymorphism annotation (Figure 9) and diversity assessment (Figure 4; Figure 5) revealed that the allelic diversity in genic regions of BNF key genes and potential BNF key genes in populations of red clover is sufficient, satisfying that prerequisite for high phenotype variability and, ultimately, BNF selection. For the candidate genes in Panel 1 and 2, expected and observed heterozygosity was calculated. In the candidate genes in Panel 1, no obvious differences between expected and observed heterozygosity were found. We assume that the analyzed plants do not deviate from Hardy–Weinberg equilibrium in the studied genes in Panel 1. In Panel 2, the difference between the expected and observed heterozygosity was found in two of the candidate genes sequences (Tp_33338, Tp_84). We can conclude that these genes do not meet the assumptions of Hardy–Weinberg equilibrium, especially the assumption that the genes are not under selection. These two genes may be subject to selection; however, this selection does not correspond to BNF rate because variants in these genes are not associated with BNF rate. Nevertheless, the specific function of this genes should be checked by gene function analysis. In addition, these genes have a low level of diversity and a low number of polymorphisms.

The discovery-driven approach of the genome-wide association study complemented the results gained by the hypothesis-driven approach of target sequencing of candidate genes. This exploratory analysis of tens of the populations using genome-wide association studies was not robust enough to clearly identify causal genes, but the results could be valuable for a breeding purpose. Although our dataset was not capable of comparing the genome-wide association studies with hundreds of samples, it was sufficient to reveal potentially associated alleles with a large effect on complex traits. Rather than finding new genes in the BNF process, our study focused on highlighting loci in the red clover genome that are potentially beneficial for BNF, and which should be selected as fixed in starting plant material for breeding new high-BNF rate varieties. On the other hand, the associated alleles of the candidate genes should be used for fine-tuning of the BNF rate red clover phenotype. Moreover, the relevance of an association signal is supported by the location of some variants in the vicinity of a gene that potentially has a role in the BNF process. In our case, we detected two significantly associated SNPs and one InDel mapped on linkage groups (Supplementary Table S3). The first associated SNP on LG4 is linked with the gene for auxin response factor and sulfotransferase. Auxin response factors are among the regulators of auxin response genes, and they play roles in various processes of plant growth and development [81]. According to Breakspear et al. [82], auxin is involved through its regulation of cell-wall remodeling in the initiation of rhizobial infection and growth of infection thread. The role of sulfotransferases is potentially connected to nitrogen fixation. Sulfotransferases enable the transfer of a sulfuryl group from a donor to an acceptor. The nitrogenase consists of two proteins, dinitrogenase reductase (Fe protein) and dinitrogenase (MoFe protein), whose structures are rich in sulfur, thus indicating that this element could be limiting in rhizobial symbiosis. Sulfur is also a part of the amino acids cysteine and methionine, and nodules contain a cysteine-rich protein, ferredoxin, which operates as an electron transporter and donates electrons to nitrogenase. Sulfur deficiency in nodulated legumes negatively affects nodulation, causing reduction in nodule number and in nodule mass per unit root length. This directly inhibits N fixation and alters the nodule metabolism. A sufficient sulfur supply contributes to increased nodulation and symbiotic nitrogen fixation [67,83]. Sulfate intake is provided by symbiotic sulfate transporters (SST), and the sulfate is reduced to organic sulfide. The symbiotic function of sulfur in the bacteroid is the sulfation of Nod factors and of cell-surface polysaccharides. The process is catalyzed by the sulfotransferase activity of NodH [83,84].

The second associated SNP on LG4 is placed near genes for ethylene-responsive transcription factor 3 (*ERF3*). The *ERF3* gene belongs to the AP2/ERF superfamily of transcription factors [62,63], and it plays a key role in crown root development and elongation. Through its interaction with cytokinin-responsive gene *RR2* from type-A RR genes, ERF3 acts as a repressor of cytokinin signaling that results in crown root initiation. In the crown root meristem, a WUSCHEL-related homeobox gene (*WOX11*) is expressed and it binds to the complex RR2/ERF3. This process leads to inhibition of ERF3 and RR2 and results in increased cytokinin signaling and crown root elongation [85].

The associated InDel on LG1 is near several genes for bidirectional amino-acid transporter 1 (BAT1). BAT1 serves as a transmembrane protein that transports amino acids in both directions through the plasma membrane. This process is necessary for amino-acid transport between xylem and phloem [86]. In the process of BNF, the nitrogen is reduced to ammonia and, using glutamate synthetase, it is incorporated into glutamate [87]. According to Dündar and Bush [86], glutamate, together with amino acids such as alanine, arginine, and lysine, is transported by BAT1.

In order to estimate the strength of the connection between genetic polymorphism variance and ^15^N BNF rate phenotype variance, we estimated marker-based narrow-sense heritability. We estimated that 84.7% of phenotypic variance is due to additive genetic effects expressed in genotypic polymorphism data. The high level of BNF rate heritability corresponds to the high levels of heritability mentioned in earlier results [10,50], and it predetermines associated polymorphisms to be good genetic markers for the prospective genomic selection of a new variety with high BNF rate that is based on the assessed collection of populations.

In conclusion, knowledge of genotype–phenotype associations led to a deeper understanding of how genotype leads to phenotype, and DNA markers could be developed based on characterized gene polymorphisms. Due to the statistical approach of association studies, functional validation of candidate polymorphisms will be essential for their implementation. SNP microarrays and InDel-specific markers will be designed for genotyping and co-segregation studies in red clover. Both provide an important resource in the form of beneficial alleles for efficient marker-assisted selection and application in red clover breeding for improved nitrogen fixation capacity. To link theory with practice, the results of this study will be used as input molecular markers for a high-throughput genotyping platform using a DNA microarray. The DNA microarray platform will be used as a tool in BNF rate breeding program of red clover. In particular, the associated polymorphisms from the population genome-wide association study could be used as markers for the pre-selection of appropriate input red clover populations for breeding on BNF efficiency. On the other hand, the associated variants from the candidate genes panels will be used to fix the beneficial alleles of BNF candidate genes in breeding populations. Finally, the association level of selected polymorphisms will have to be validated in practice using the first generation of the mentioned DNA microarray before it can be implemented in a real red clover BNF breeding program.

## 4. Materials and Methods

### 4.1. Plant Materials

Three plant sets and one plant population for BNF rate evaluation were prepared, the former for ARA and the latter for the natural ^15^N-abundance method. Sets 1, 2, and 3 of plants were grown in 2017, 2018, and 2019, respectively: in 2017, 647 plants of four diploid (Start, Vltavín, Columbia, Global) and four tetraploid (Tatra, Tempus, Kvarta, HJRH) accessions; in 2018, 401 plants of four tetraploid accessions (Nodula, Gregale, Atlantis, Tempus); and, in 2019, 378 plants as offspring of 16 parents selected in Set 1. In total, 1426 plants were grown and the number of plants per accession varied between years. In Sets 1 and 2, higher numbers of plants per accession were grown to assess intrapopulation BNF diversity and to find high- and low-BNF rate plants among broad input populations. In Set 3, we used a smaller number of plants per accession to assess how real selection works.

In order to the BNF evaluation by natural ^15^N-abundance approach, population samples consisted of 91 diploid accessions and originated from the Czech core collection of *T. pratense* within the Czech national seed bank, which is maintained by the Crop Research Institute (Prague, Czech Republic). The list contained varieties and wild accessions. *Galega orientalis* Lam. uninoculated by *Neorhizobium galegae* and non-nitrogen symbiotic plants *Malva verticillata* L. were used as controls. Red clover accessions and their characteristics are summarized in Supplementary Table S2.

### 4.2. Growth Conditions and Evaluation of Nitrogen Fixation by Acetylene Reduction Assay

The red clover seeds were scarified and germinated on wet perlite. Sprouted seeds were planted in individual pots filled with perlite and inoculated with rhizobia by adding 1 mL of *Rhizobium leguminosarum* bv. *trifolii* inoculum, which was provided by the Crop Research Institute (Prague, Czech Republic). Different rhizobia strains were applied for diploid and tetraploid varieties as recommended by the collection’s curator. Plants were grown hydroponically in a greenhouse within individual pots filled with perlite. They were watered with a nutrient solution containing 870 mg/L K_2_HPO_4_, 135 mg/L FeCl_3_∙6H_2_O, 735 mg/L CaCl_2_∙2H_2_O, 246 mg/L MgSO_4_∙7H_2_O, 0.123 mg/L Na_2_MoO_4_∙H_2_O, 0.486 mg/L H_3_BO_3_, 0.055 mg/L CuSO_4_∙5H_2_O, 0.25 mg/L MnCl_2_∙4H_2_O, and 0.06 mg/L ZnSO_4_∙7H_2_O. No nitrogen was supplied exogenously, and the pH was 6.5–6.8. The solution was replenished as necessary and exchanged once a week. ARA was used for evaluating the efficiency of nitrogen fixation in individual plants through analyzing nitrogenase activity [56]. ARA was carried out approximately 100 days after sowing. The results were expressed as concentration of ethylene C_E_ (μmol/mL) in a jar after 0.5 h of incubation.

### 4.3. Evaluation of Nitrogen Fixation by Natural ^15^N-Abundance Method

The 15 bulked plants per accession were grown in pots with soil from local field with red clover. The plants were sampled at the beginning of flowering of early accessions. The nitrogen (N) and carbon (C) concentrations and their isotopic compositions in red clover shoots (ground to a fine powder using a Retsch MM200 ball mill, sample weights 3–4 mg, packed in tin capsules) were measured using a Flash EA 2000 elemental analyzer coupled with a Delta V Advantage isotope ratio mass spectrometer (both Thermo Scientific, Waltham MA, USA). Elemental composition was calibrated using certified standards from Elemental Microanalysis (Okehampton, UK). Isotopic composition was assessed by comparison with certified standards from the International Atomic Energy Agency (Vienna, Austria).

### 4.4. Selection of Candidate Genes and Procedure of Targeted Sequencing

Selection of candidate genes was carried out based on the annotated genome of the model legume *M. truncatula*. The genes essential for the nodulation process and nitrogen fixation were chosen for sequencing. Overall, 17 and 69 chosen candidate genes from Panels 1 and 2, respectively, included genes for transcription factors, receptor-like kinases (*RLK*), leghemoglobins, and cytokinin receptors (Supplementary Table S1). Many of these genes were functionally characterized for their roles in the nitrogen fixation process. Sequences of these genes extracted from the GeneBank database (https://www.ncbi.nlm.nih.gov) were aligned to the genome sequence of *T. pratense* variety Tatra [34] using BLAST+ (ver. 2.8.1, [88]). Sequences with highest similarity (>90%) were chosen for further analysis. The Panel 1 span was 95,000 bp and that of Panel 2 was 98,464 bp of the red clover genome.

Forty-eight and 50 plants from Sets 1 and 2, respectively, with the most contrasting BNF values were used for SeqCap. One hundred milligrams of fresh leaves were collected, and flash-frozen in liquid nitrogen. DNA was isolated using a DNeasy Plant Mini Kit (Qiagen, Germany) according to the manufacturer’s protocol and following the cetyl trimethylammonium bromide (CTAB) method [89]. DNA quality was checked on a 3% agarose gel, and DNA concentration was quantified by NanoDrop 2000c spectrophotometer (Thermo Fisher Scientific, USA) and by a Qubit fluorometer (Invitrogen/Thermo Fisher Scientific, USA).

Probe design was performed with Roche NimbleGen’s custom probe design pipeline (Roche Diagnostic, USA; http://www.nimblegen.com/products/seqcap/ez/designs/). Two gene panels were designed (Supplementary Table S1). Gene Panel 1 spanned 95 kbp of the selected genomic sequences, including the 17 candidate genes. Gene Panel 2 spanned 99.5 kbp and the 69 genes. Forty-eight and 50 DNA samples were sequenced for Panels 1 and 2, respectively. Libraries of both panels were prepared using the SeqCap EZ HyperCap procedure (Roche Diagnostic, USA) while following the manufacturer’s instructions, and the libraries were sequenced for 150-bp reads with paired-end sequencing on a NextSeq 500 sequencer (Illumina, San Diego, CA, USA). Library preparation and sequencing were performed at Core Facility Genomics CEITEC MU (Brno, Czech Republic).

### 4.5. ddRADseq Library Preparation and Sequence Processing of T. pratense Population Set

Ninety-six batch samples of 15 plants per sample (Supplementary Table S2) were processed together into one final ddRADseq library. Library preparation followed a slightly modified protocol by Peterson et al. [40]. Three hundred nanograms of genomic DNA from each population was digested with two restriction enzymes, *Sph*I and *MluC*I, in one 30-μL reaction. P1 and P2 “flex” adapters were ligated in a 40-µL reaction with 100 ng of the digestion product. The total volume of 48 ligation products differing in adapter barcode were pooled together into a “sublibrary”, and two sublibraries in total were prepared. The order of samples was randomized between and within sublibraries. Automated size selection of a fraction of 220–320 bp separately from each sublibrary was performed on the Pippin Prep laboratory platform using a Pippin Prep 2010 kit (Sage Science, Beverly, MA, USA). PCR amplification with primers bearing the multiplexing indices and Illumina flow cell annealing regions was done in several 50-μL reactions (separately for each sublibrary). PCR products were purified on AMPure XP beads and combined in equimolar ratios to compose the final library. Sequencing was performed using 125-bp paired-end reads on a HiSeq 2500 (Illumina) at the EMBL Genomic Core Facility, Heidelberg, Germany.

### 4.6. Bioinformatic Analysis

Basic characteristics of the reads obtained were reviewed in FastQC v0.10.1 [90]. Barcode sorting was performed in process_radtags, a pipeline component of Stacks v2.3 [91]. A reference-based strategy was used for assembling the targeted sequences and ddRADseq sequences obtained. Reads were firstly qualitatively filtered and trimmed using Trimmomatic v0.38 [92], and then aligned onto the genomes of *T. pratense* [34,35] reference genomes with Milvus and Tatra varieties using the BWA-MEM algorithm from BWA v0.7.17 assembler [93]. Sequence data from target sequencing were randomly downsampled to 150× coverage. GATK (Genome Analysis Toolkit) v4.1.0.0 [94] was used for base quality score recalibration and performing SNP and InDel variant calling across samples of target sequencing and ddRADseq population genotyping as well. Variants were filtered using standard hard filtering parameters according to GATK Best Practices recommendations [95,96].

In order to express genotypes information of bulked samples in ddRADseq population genotyping, continuous numerical genotypes were computed as frequencies of allelic depth counted from allelic depths and read depth in variant positions. The polymorphisms that were identified with a maximum of 50% missing information and polymorphisms that were polymorphic in more than 5% of called population numeric genotypes were used for the analysis. Missing population genotypes were imputed before association analysis as means of continuous numerical genotypes of the variants.

For target sequencing, Panel 1 and 2 genotypes were called in diploid and tetraploid states. All variants from candidate gene panels sequencing and also from ddRADseq genotyping were annotated using Variant Effect Predictor (VEP) [97]. Called final variants of Panel 1, Panel 2, and the population ddRADseq genotype are stored and presented in Supplementary Table S4.

### 4.7. Statistical Analysis

Results of ARA were expressed as ethylene molar concentration (C_E_) values that were computed from ethylene peak area in accordance with Unkovich et al. [52]. The C_E_ value was standardized to *Z*-score within measuring sets in order to compare BNF rate among different sets. Differences in nitrogen fixation rate measured using ARA among different populations were tested with the nonparametric Kruskal–Wallis test and subsequent nonparametric post hoc comparisons.

Polymorphism diversity level was expressed as expected heterozygosity (Hs). This was computed as if the species were diploid, because it is also appropriate for diversity comparison for polyploid cases [98]. In order to assess if the genes meet the assumptions of Hardy–Weinberg equilibrium, observed heterozygosity for candidate genes was calculated as well. To test differences between expected heterozygosity and observed heterozygosity, we used a Mann–Whitney U test in R. Genetic diversity pattern was assessed by principal component analysis using the pcaMethods R package [99].

The association analyses of variants from candidate genes in Panels 1 and 2 were conducted using the mixed linear model algorithm [100] in GAPIT in R.

The genome-wide association study for variants from population genotyping were conducted using the statistical method FarmCPU [45], and estimation of marker-based heritability was performed in GAPIT in R [101]. The significance threshold was set to the false discovery rate-adjusted *p*-value of 0.05 using the Benjamini–Hochberg correction [46].

### 4.8. Validation of Selected InDel Polymorphisms

For validation, 10 InDels for six different candidate genes (Supplementary Table S6) from Panel 1 were chosen. Genotypes used for validation are given in Supplementary Table S6. Validation was performed by means of allele-specific PCR and 3% agarose gel electrophoresis; surrounding primers were designed for InDels longer than 50 bp, and the products were clearly distinguished according to the length of the PCR products. For InDels shorter than 50 bp, one of the primers hybridized to the sequence of the InDels and the other one matched the sequence adjacent to the InDel. In this case, PCR products were only visible if the genotypes contained the desired InDels. Specificity of the designed primers was verified using BLAST+ (ver. 2.8.1, [88]) with *T. pratense* var. Tatra [34] as a database.

## 5. Conclusions

Red clover plants with high BNF rate contribute more to the accumulation of biogenic nitrogen in the soil to improve sustainability in agriculture. We performed genome-wide and targeted association studies and described phenotypic and genotypic variation of BNF in red clover, which allowed finding key candidate genes responsible for this complex polygenic trait. We identified polymorphisms in key genes strongly associated with BNF rate: *EFD*, which negatively regulates the nodulation process and positively influences cell differentiation in the late stages of nodulation, and *MOT1*, which is responsible for molybdate intake of nodule cells. Our population genotyping data confirmed polymorphisms strongly associated with BNF and located near the genes for auxin response factor, which regulates the cell-wall remodeling, and sulfotransferase involved in the process of sulfur metabolism, and also near *ERF3* regulating the crown root development and *BAT1* ensuring bidirectional transport of amino acids between xylem and phloem. 

In comparison with conventional breeding of red clover, breeding based on genomic data can be effective in dealing with complex polygenic traits like BNF. It can help to identify and select additive genes or beneficial recessive alleles even at tetraploid varieties of cross-pollinating species. Because of the statistical approach of association studies, functional validation of those candidate polymorphisms found will be essential for confirming the biological importance of the alleles identified to be beneficial for efficient red clover selection and breeding for improved nitrogen fixation capacity. The practical outcome of this study will provide input molecular markers for the high-throughput DNA microarray genotyping platform that will be used for breeding of new red clover varieties with higher BNF rate.

## Figures and Tables

**Table 1:** Statistically significant differences of nitrogen fixation capacity within three evaluated sets of red clover plants using acetylene reduction assay.

Plant Set	*p*-Value ^1^	Different Pairs of Accessions ^2^
1	3.413 × 10^−6^	Columbia17-HJRH17, Columbia17-Kvarta17
2	1.151 × 10^−6^	Nodula18-Gregale18, Nodula18-Tempus18
3	2.2 × 10^−16^	Kvarta17.73-Start17.58, Kvarta17.81-Start17.58, Start17.25-Start17.58, Start17.39-Start17.50, Start17.46-Start17.58, Start17.46-Tempus17.5, Start17.50-Start17.55, Start17.50-Start17.58, Start17.50-Tempus17.4, Start17.50-Tempus17.5, Start17.50-Tempus17.57, Start17.52-Start17.58, Start17.58-Tatra17.16, Start17.58-Tempus17.27, Start17.58-Tempus17.40, Tatra17.16-Tempus17.5, Tempus17.40-Tempus17.5

^1^ Kruskal–Wallis test; ^2^ statistical significance for *p* < 0.01.

## Supplementary Materials

Supplementary materials can be found at https://www.mdpi.com/1422-0067/20/21/5470/s1. Supplementary Figure S1. targeted sequences coverage. Supplementary Figure S2. InDel validation. Supplementary Figure S3. correlation of phenotype and diversity pattern. Supplementary Table S1. Targeted sequence. Supplementary Table S2. Plant material. Supplementary Table S3. Associated polymorphisms. Supplementary Table S4. Genotypic tables. Supplementary Table S5. Polymorphisms indel validation.

## Abbreviations

AP2	APETALA2
ARA	Acetylene reduction assay
ATP	Adenosine triphosphate
BNF	Biological fixation of atmospheric nitrogen
C_E_	Ethylene molar concentration
CRE	Cytokinin response 1
CTAB	Cetyl trimethylammonium bromide
ddRADseq	Double-digest RAD sequencing
DNF2	Defective in nitrogen fixation 2
EFD	Ethylene response factor required for nodule differentiation
ERF	Ethylene response factor
ERF3	Ethylene-responsive transcription factor 3
FLOT	Flotillin
GATK	Genome Analysis Toolkit
Hs	Expected heterozygosity
InDel	Insertion/deletions
LG	Linkage group
MOT1	Molybdate transporter 1
ModABC	ATP-binding cassette transporter involved in molybdate transport
Mt	*Medicago truncatula*
NFP	Nod factor perception
NF-YC2	Nuclear transcription factor Y subunit C2
NGS	Next-generation sequencing
Nod factors	Nodulation factors
PC	Principal component
PCA	Principal component analysis
PNO1	partner of NOB1-like
RLK	Receptor-like kinases
RR2	Cytokinin responsive gene
SeqCap	Hybridization-based sequence capture
SNP	Single-nucleotide polymorphism
SST	Symbiotic sulfate transporter
Tp	*Trifolium pratense*
VEP	Variant effect predictor
WOX11	WUSCHEL-related homeobox gene
