# Supplementary material for: Allelic Variants for Candidate Nitrogen Fixation Genes Revealed by Sequencing in Red Clover (Trifolium pratense L.)
Source: Int J Mol Sci. 2019 Nov 2;20(21):5470. doi: 10.3390/ijms20215470 (PMC6862357; doi:10.3390/ijms20215470)
Supplement: Supplementary file 1 [file ijms-20-05470-s001.zip › Supplementary/Supplementary_figures.pptx]

## Slide 1
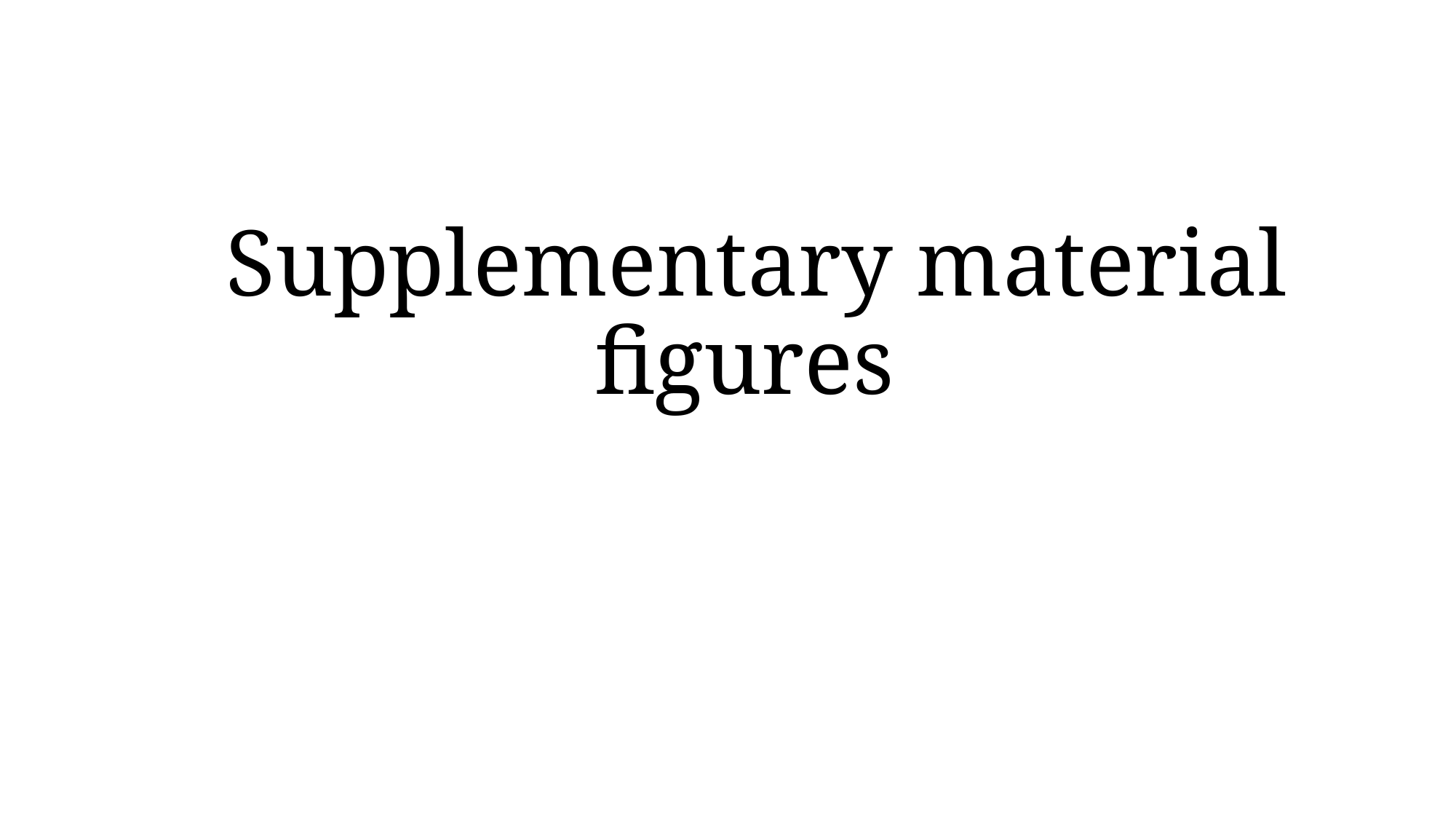

# Supplementary material figures

## Slide 2
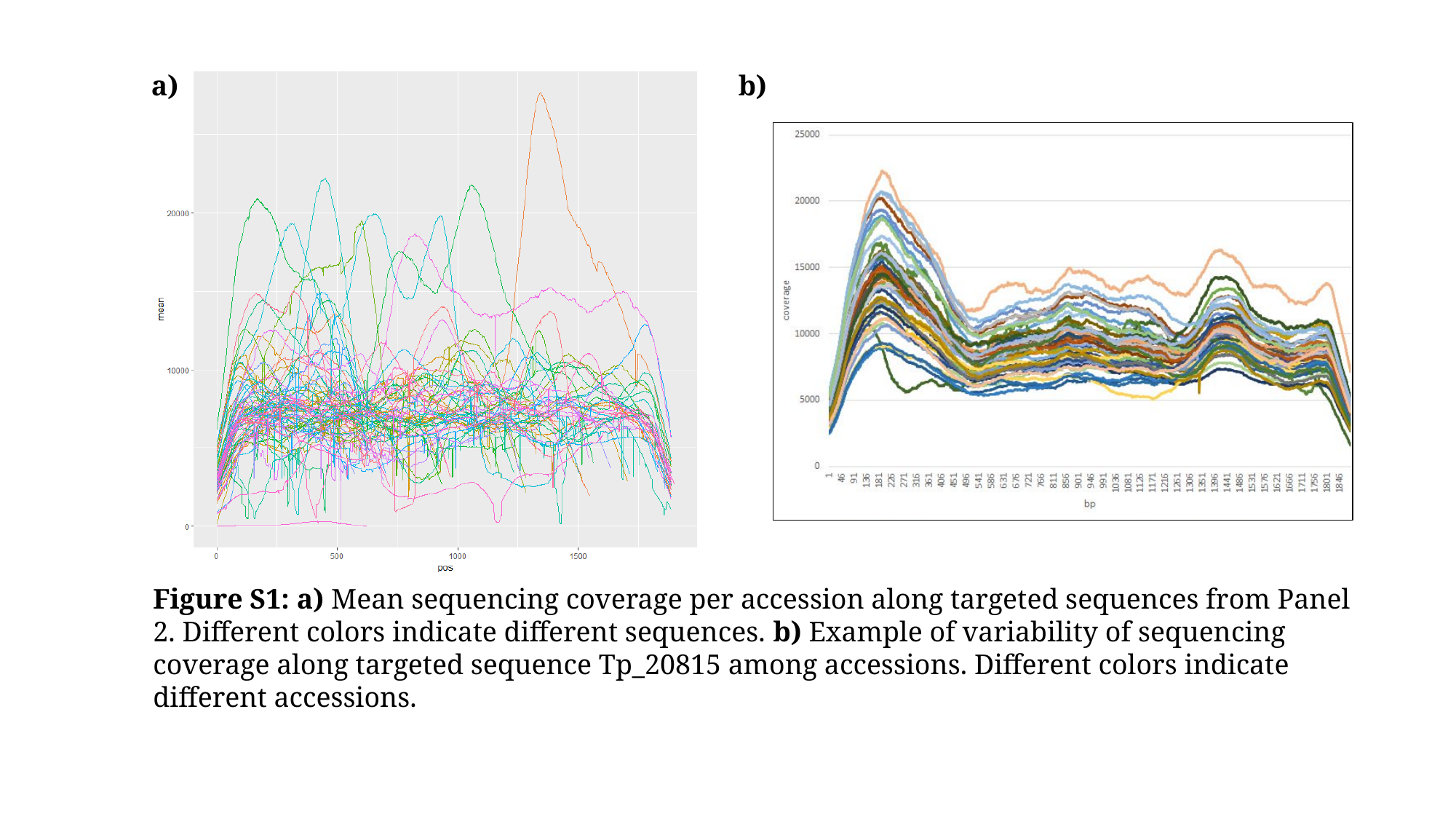

a)
b)
Figure S1: a) Mean sequencing coverage per accession along targeted sequences from Panel 2. Different colors indicate different sequences. b) Example of variability of sequencing coverage along targeted sequence Tp_20815 among accessions. Different colors indicate different accessions.

## Slide 3
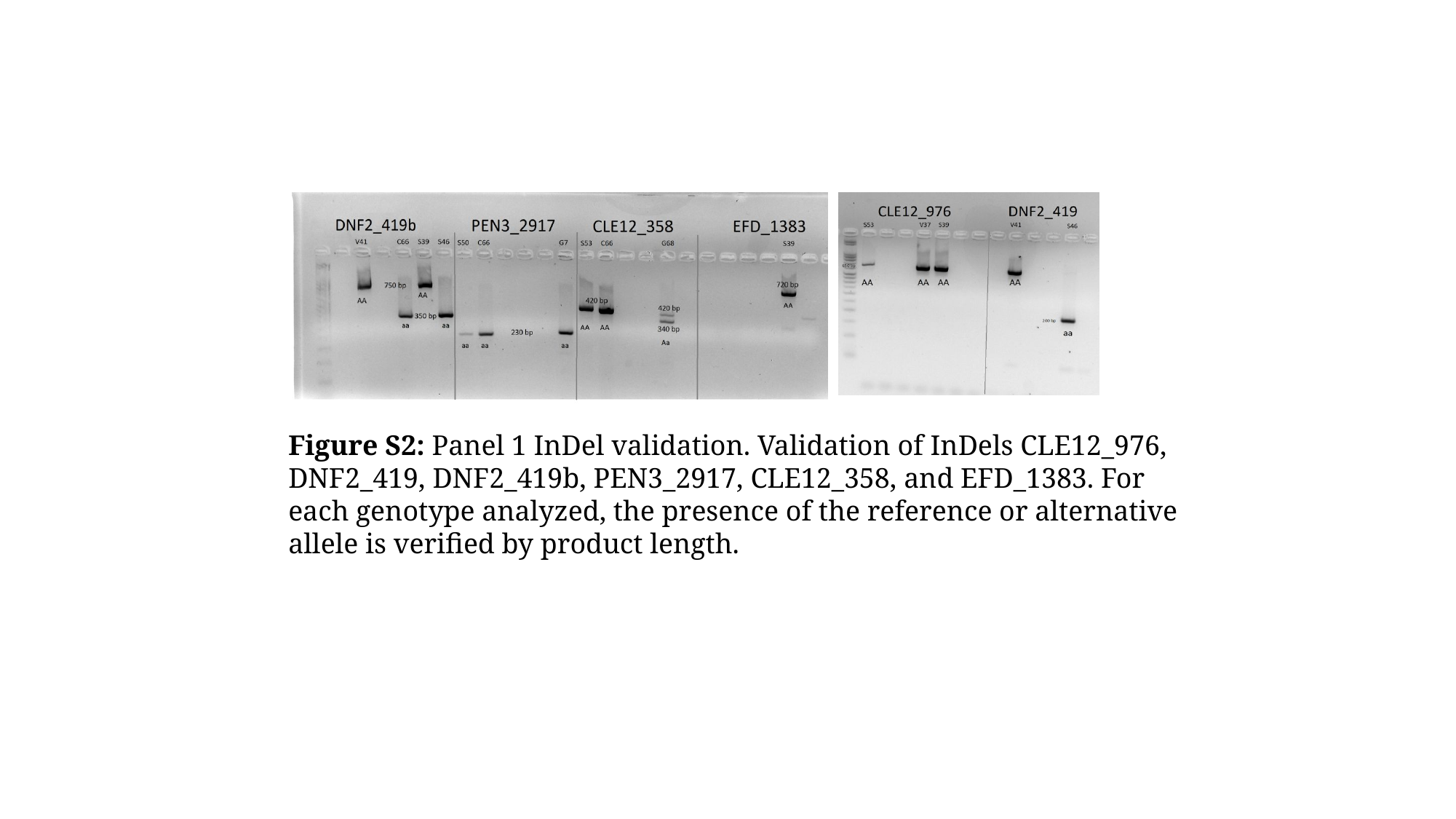

Figure S2: Panel 1 InDel validation. Validation of InDels CLE12_976, DNF2_419, DNF2_419b, PEN3_2917, CLE12_358, and EFD_1383. For each genotype analyzed, the presence of the reference or alternative allele is verified by product length.

## Slide 4
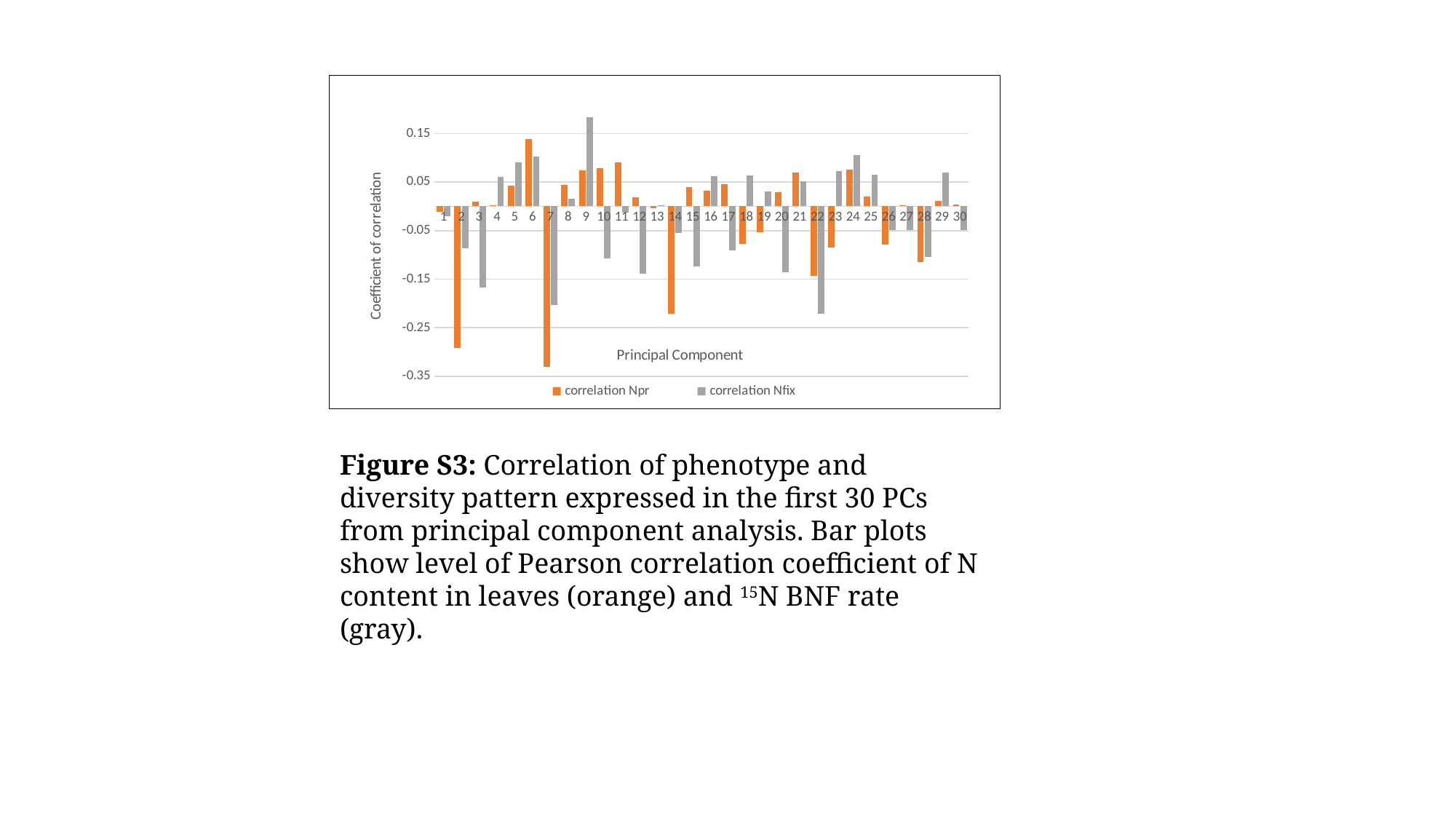

### Chart
| Category | correlation Npr | correlation Nfix |
|---|---|---|Figure S3: Correlation of phenotype and diversity pattern expressed in the first 30 PCs from principal component analysis. Bar plots show level of Pearson correlation coefficient of N content in leaves (orange) and 15N BNF rate (gray).
